# Effect of Post-Fabricated Aging on Microstructure and Mechanical Properties in Underwater Friction Stir Additive Manufacturing of Al–Zn–Mg–Cu Alloy

**DOI:** 10.3390/ma15093368

**Published:** 2022-05-07

**Authors:** Ying Li, Changshu He, Jingxun Wei, Zhiqiang Zhang, Ni Tian, Gaowu Qin, Xiang Zhao

**Affiliations:** 1 School of Materials Science & Engineering, Northeastern University, Shenyang 110819, China; liying3273@163.com (Y.L.); jingxunwei@foxmail.com (J.W.); lnkdzzq@126.com (Z.Z.); tiann@atm.neu.edu.cn (N.T.); qingw@smm.neu.edu.cn (G.Q.); zhaox@mail.neu.edu.cn (X.Z.); 2 Key Laboratory for Anisotropy and Texture of Materials, Northeastern University, Shenyang 110819, China; 3 Research Center for Metallic Wires, Northeastern University, Shenyang 110819, China

**Keywords:** friction stir additive manufacturing, Al–Zn–Mg–Cu alloy, aging, precipitation hardening, microstructure, mechanical property

## Abstract

The fabricated Al–Zn–Mg–Cu alloy build has low mechanical properties due to the dissolution of strengthening precipitates back into the matrix during friction stir additive manufacturing (FSAM). Post-fabricated aging was considered an effective approach to improve the mechanical performance of the build. In this study, various post-fabricated aging treatments were applied in the underwater FSAM of Al–7.5 Zn–1.85 Mg–1.3 Cu–0.135 Zr alloy. The effect of the post-fabricated aging on the microstructure, microhardness, and local tensile properties of the build was investigated. The results indicated that over-aging occurred in the low hardness zone (LHZ) of the build after artificial aging at 120 °C for 24 h as the high density of grain boundaries, subgrain boundaries, dislocations, and Al_3_Zr particles facilitated the precipitation. Low-temperature aging treatment can effectively avoid the over-aging problem. After aging at 100 °C for 48 h, the average microhardness value of the build reached 178 HV; the yield strength of the LHZ and high hardness zone (HHZ) was 453 MPa and 463 MPa, respectively; and the ultimate tensile strength of the LHZ and HHZ increased to 504 MPa and 523 MPa, respectively.

## 1. Introduction

Additive manufacturing (AM), also known as 3D printing, is a technique for the layer-by-layer fabrication of objects using computer-aided design (CAD) [1,2]. AM technologies using high-intensity energy sources such as the laser, electron beam, and wire arc have been widely investigated over the last three decades owing to their advantages such as high production flexibility and high efficiency [3,4,5]. However, it is challenging for the fusion-based AM to fabricate some light-alloy components, such as Al and Mg alloys, due to the generation of various solidification-related defects such as solidification cracking and porosity [6,7]. Thus, solid-state AM has received increasing attention [8,9,10].

Friction stir additive manufacturing (FSAM) is one of the innovative solid-state AM techniques based on friction stir welding (FSW) [11]. As the FSW technology in the joining of Al and Mg alloys is increasingly maturing, FSAM technology can be potentially used to fabricate Al and Mg alloy components. This technique has been recently used to fabricate 5083 [12], 6061 [13], 2195 [14,15], 7075 [16], 7N01 [17,18], and 7A04 [19] Al alloys and the WE43 [20] and AZ31 [21] Mg alloys. The hot topics of this research mainly focus on the optimization of the formation, microstructure, and mechanical performance of the build.

Al–Zn–Mg–Cu (7xxx) alloy is widely employed in the aerospace industry owing to its high strength-to-weight ratio, and its yield strength (YS) exceeded 500 MPa and its ultimate tensile strength (UTS) reached 580 MPa after aging, according to Scharifi et al. [22,23]. Its strengthening mechanism mainly depends on precipitation strengthening. The precipitation sequence of the Al–Zn–Mg–Cu alloy is generally recognized as follows: supersaturated solid solution-Guinier–Preston zone (GP zone)-semi-coherent η′ phase-equilibrium η phase (MgZn_2_) [24,25]. The fine and high-density precipitates of the GP zones and the ηʹ phases are considered to be the primary strengthening agents [26]. Similarly, precipitate characterization is also a crucial factor when evaluating the mechanical properties of the Al–Zn–Mg–Cu alloy build fabricated by the FSAM.

During the FSAMed Al–Zn–Mg–Cu alloy process, fine equiaxed grains are produced due to the severe plastic deformation and high temperature. Meanwhile, during the heating process, the second particles are broken and dissolved into the matrix, and then may precipitate again during the cooling stage [16]. Furthermore, variable thermal exposure is experienced in the different layers of the fabricated build due to the layer-by-layer additive process characteristics [27,28]. The closer a layer is to the bottom of the build, the more thermal cycles it experiences. Coarsening of the grains and precipitates is prone to occur, which would definitely alter the mechanical properties of the build. Mao [16] and He [17] discovered that the microhardness and the tensile strength of the 7xxx aluminum alloy build decreased from top to bottom, which was attributed to the grain growth and precipitate coarsening induced by the multipass thermal cycling. In-process water cooling in the FSAM has been proven as an effective approach for solving the abovementioned macro-softening problem. Relatively uniform microstructures and mechanical properties along the building direction of the 7N01 aluminum alloy build were obtained via underwater FSAM [18]. In the cooling stage of the FSAM process and the subsequent reheating process, water cooling increased the cooling rate and reduced the precipitation, resulting in a high degree of supersaturation. However, the mechanical properties of the fabricated build with the supersaturated state were low because the strengthening precipitates dissolved back into the matrix. Therefore, post-fabricated aging was considered to enhance the mechanical performance of the build. It has been reported that the ultimate tensile strength of the water-cooled 7N01 aluminum alloy builds reached 400 MPa after 90 days of natural aging, which is slightly higher than that of the base metal in T4 temper (392 MPa) [18]. However, when the post-fabricated aging treatment was applied in the underwater FSAMed 7A04 aluminum alloy, various microhardness recovery degrees were present in the build. After aging at 120 °C for 24 h, the microhardness enhancement in the bottom of each stir zone of the build was lower than that of other regions, which was mainly attributed to the high density of the Mg(Zn,Cu,Al)_2_ phase precipitation induced by the fine grains and high density of subgrains and dislocations in this region [19]. This loss of microhardness and reduction in tensile strength of 7xxx aluminum alloy has also been reported in the FSW joint [29,30,31]. Efforts to recover post-processing strength are achieved through solution heat treatment combined with subsequent artificial aging [32]. During the solution treatment, the soluble phase can be re-dissolved into the matrix [33]. The artificial aging process caused the re-precipitation of finer precipitates, resulting in an increase in the tensile properties [34]. However, the high-temperature solution treatment is challenging for some large-scale builds. Additionally, the fabricated builds are prone to distortion during the solution treatment, and abnormal grain growth in the stir zone has been reported [35,36,37]. Therefore, to improve the mechanical properties, it is necessary to select appropriate post-fabricated aging without solution treatment applied on the fabricated build.

In this study, several post-fabricated aging treatments are applied in the underwater FSAM of Al–7.5 Zn–1.85 Mg–1.3 Cu–0.135 Zr alloy. The influences of post-fabricated aging on the microstructure, microhardness, and tensile properties of the build are investigated.

## 2. Materials and Methods

### 2.1. Materials and Friction Stir Additive Manufacturing Processing

**Figure 1:**
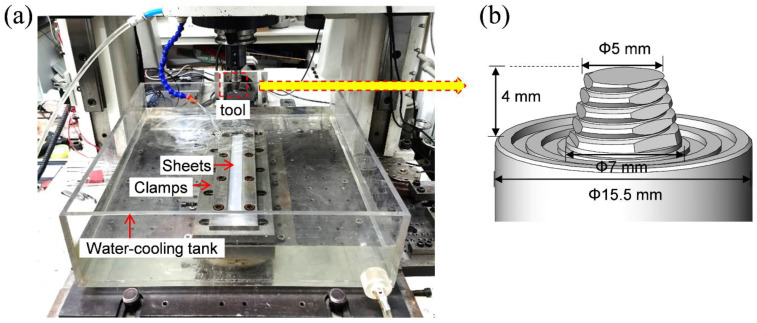
(**a**) Experimental setup of the underwater FSAM; (**b**) dimensions of the tool used in this study.

The hot-rolled Al–7.5 Zn–1.85 Mg–1.3 Cu–0.135 Zr alloy sheets with a size of 300 × 25 × 3.5 mm after solution treatment at 465 °C for 20 min and 480 °C for 30 min were used for underwater FSAM. The chemical composition of the base metal is shown in Table 1. Figure 1 shows the experimental setup of the underwater FSAM and the dimensions of the tool used in this study. The tool rotation and traveling speeds during the FSAM process were 700 r/min and 160 mm/min, respectively. The cooling water temperature was set to 15 °C in an external cyclic water-cooling tank. The top surface of the build was submerged in the water at a depth of 15 mm.

After the underwater FSAM, samples were taken from the build for natural aging for 7 days (NA-7 d), artificial aging at 120 °C for 24 h (120 °C × 24 h), 100 °C for 24 h (100 °C × 24 h), 48 h (100 °C × 48 h), 72 h (100 °C × 72 h), 80 °C for 24 h (80 °C × 24 h), 48 h (80 °C × 48 h), and 72 h (100 °C × 72 h). Then, the microhardness and tensile properties of the builds in different aging states were tested, and the microstructure was observed.

### 2.2. Characterization of Properties and Microstructure

The microhardness distributions on the cross-section of the builds in different aging states were tested along the building direction using a Vickers microhardness tester (401-MVD, Wolpert Wilson Instruments) with a 100 g load and a 0.5 mm interval.

The tensile samples were taken from the builds in different aging states along the traveling direction. The tensile properties of the samples were tested at a constant crosshead speed of 1 mm/min using a 100 kN Shimadzu AG-Xplus electronic universal testing machine at room temperature.

The characteristics of the strengthening precipitates of the builds in different aging states were analyzed via transmission electron microscopy (TEM, JEOL JEM-2100F) and high-resolution TEM (HRTEM). Additionally, a scanning TEM (STEM) observation combined with the EDS test was performed. The samples were prepared by twin-jet thinning in a solution of 25 vol.% nitric acid and 75 vol.% methanol at temperatures ranging from −30 °C to −25 °C with 12 V voltage.

## 3. Results

### 3.1. Microhardness

**Figure 2:**
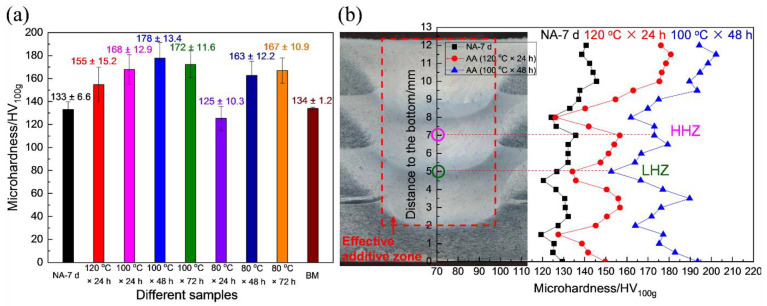
Microhardness of the builds in different aging states: (**a**) average microhardness values of the builds in different aging states; (**b**) microhardness distributions on the cross-section of the builds along the building direction in the NA-7 d, 120 °C × 24 h, and 100 °C × 48 h states.

Figure 2a shows the average microhardness of the builds in various aging treatments. They were calculated by the microhardness of the effective additive zone along the building direction. The average microhardness value of the build in the NA-7 d state was 133 HV. After artificial aging at 120 °C for 24 h, the average microhardness value increased to 155 HV. In the states of artificial aging at 100 °C, the average microhardness value increased first and then decreased as the aging time increased, and the microhardness value reached the highest value after aging for 48 h (178 HV). In the state of artificial aging at 80 °C, the average microhardness value increased with the aging time. After aging for 72 h at 80 °C, the microhardness value was 167 HV.

The typical samples of NA-7 d, 120 °C × 24 h, and 100 °C × 48 h were selected to analyze and compare their microhardness distribution along the building direction, and the results are shown in Figure 2b. In the state of NA-7 d, even though the FSAM occurred underwater, the microhardness (~140 HV) of the top layer of the build was slightly higher than those in other regions (~130 HV), which may be related to the re-stirring and re-heating that occurred during the FSAM process, resulting in the precipitation of the coarse particles. Furthermore, it was discovered that the microhardness at the bottom of each stir zone was slightly lower than those of other regions, and the low and high hardness periodically appeared along the building direction. After aging at 120 °C for 24 h, the microhardness of the low hardness zone (LHZ) was around 125 HV, and it did not significantly improve compared with that in the state of NA-7 d. However, the microhardness of the high hardness zone (HHZ) increased by 25 HV, reaching a maximum value of 155 HV. After aging at 100 °C for 48 h, the microhardness of the LHZ was around 160 HV, which increased by 40 HV compared with that in the state of NA-7 d (120 HV). Meanwhile, the microhardness of the HHZ increased by 50 HV and reached about 180 HV. The increase in the microhardness at 100 °C × 48 h was significantly higher than that at 120 °C × 24 h. Even in the LHZ, the microhardness also increased obviously after aging at 100 °C for 48 h.

### 3.2. Local Mechanical Properties

**Figure 3:**
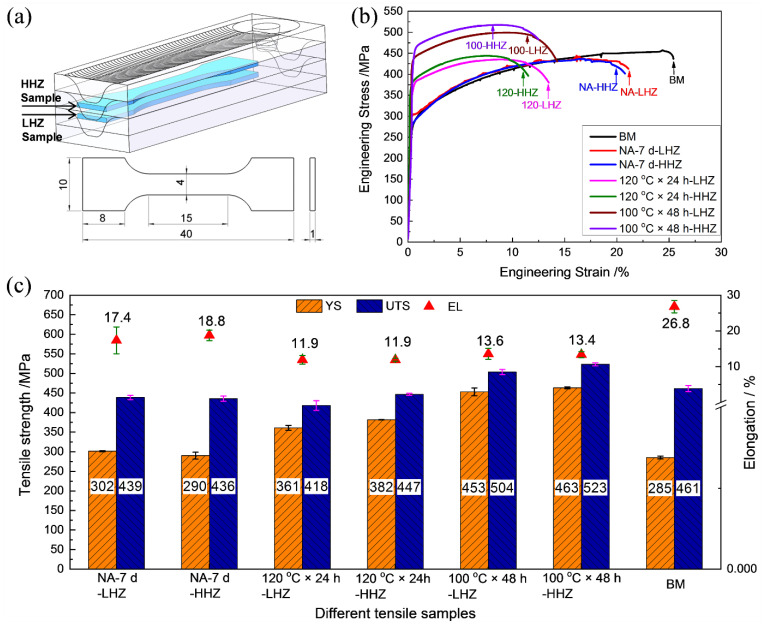
Tensile properties of the samples extracted from the low hardness zone (LHZ) and the high hardness zone (HHZ) of the builds in the NA-7 d, 120 °C × 24 h, and 100 °C × 48 h states: (**a**) schematic of the extraction position and dimension of the tensile samples (units: mm); (**b**) engineering stress–strain curves of the typical tensile samples; and (**c**) tensile strength and elongation.

According to the microhardness distribution characteristics of the builds, local tensile samples were extracted from the LHZ and HHZ, to evaluate the tensile properties in the states of NA-7 d, 120 °C × 24 h, and 100 °C × 48 h. Figure 3a shows the schematic of the extraction position and dimension of the tensile samples. Figure 3b shows the engineering stress–strain curves of the typical tensile samples. In each condition, three specimens were tested, and the average values are shown in Figure 3c. In the NA-7 d state, the yield strength (YS) and ultimate tensile strength (UTS) of the LHZ of the build were 302 MPa and 439 MPa, respectively, with an elongation (EL) of 17.4%. The YS, UTS, and EL of the HHZ were 290 MPa, 436 MPa, and 18.8%, respectively. There was no significant difference in the tensile properties of the LHZ and HHZ in the NA-7 d state. Compared with the tensile properties of the solution-treated base metal (BM), although the YS of the LHZ and HHZ was higher than that of the BM (285 MPa), the UTS and EL were lower than those of the BM (461 MPa, 26.8%). After artificial aging at 120 °C for 24 h, the YS of the LHZ (361 MPa) and HHZ (382 MPa) improved by 59 MPa and 92 MPa, respectively, in comparison with that in the NA-7 d state. However, the UTS did not change significantly, and the EL decreased to 11.9%. After artificial aging at 100 °C for 48 h, the YS of the LHZ (453 MPa) and HHZ (463 MPa) increased by 151 MPa and 173 MPa, respectively; and the UTS of the LHZ (504 MPa) and HHZ (523 MPa) increased by 65 MPa and 87 MPa, respectively, in comparison with that in the NA-7 d state. The increase in the strength of the samples aged at 100 °C for 48 h was more visible than that of the samples aged at 120 °C for 24 h, and the elongation was also higher.

Table 2 shows a comparison of tensile properties between the typical Al–Zn–Mg–Cu alloys and the Al–Zn–Mg–Cu alloy builds fabricated by additive manufacturing. Compared with the artificially aged 7085 aluminum alloy sheets that have a similar chemical composition to the base metal used in this sturdy [38,39], the tensile properties of the Al–7.5 Zn–1.85 Mg–1.3 Cu–0.135 Zr alloy build fabricated by the underwater FSAM after aging at 100 °C for 48 h in this study were slightly lower. The incomplete solid solution of the build after the underwater FSAM may be the main reason for the decrease in the tensile properties. However, compared with the builds fabricated by other fusion-based additive manufacturing, such as wire-arc additive manufacturing (WAAM) [6,7,40] and selective laser melting (SLM) [41,42], this study showed higher strength and elongation. The enhancement of tensile properties was primarily caused by the grain refinement and hot crack suppression.

### 3.3. Microstructure

**Figure 4:**
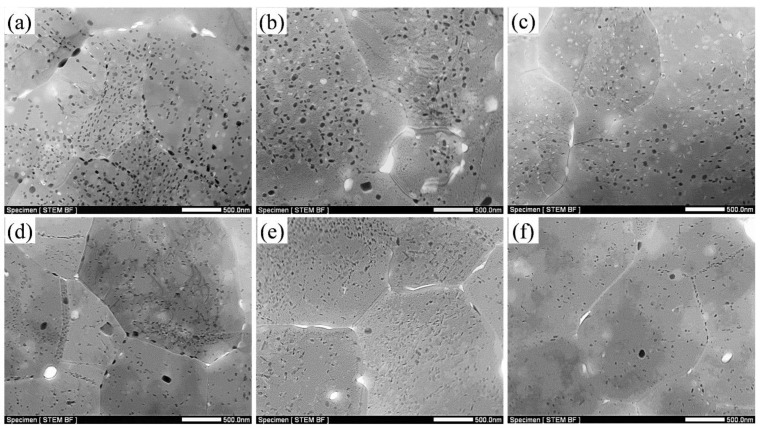
STEM images in LHZ and HHZ of the builds in the different aging states: (**a**) LHZ in the NA-7 d state; (**b**) LHZ in the 120 °C × 24 h state; (**c**) LHZ in the 100 °C × 48 h state; (**d**) HHZ in the NA-7 d state; (**e**) HHZ in the 120 °C × 24 h state; (**f**) HHZ in the 100 °C × 48 h state.

**Figure 5:**
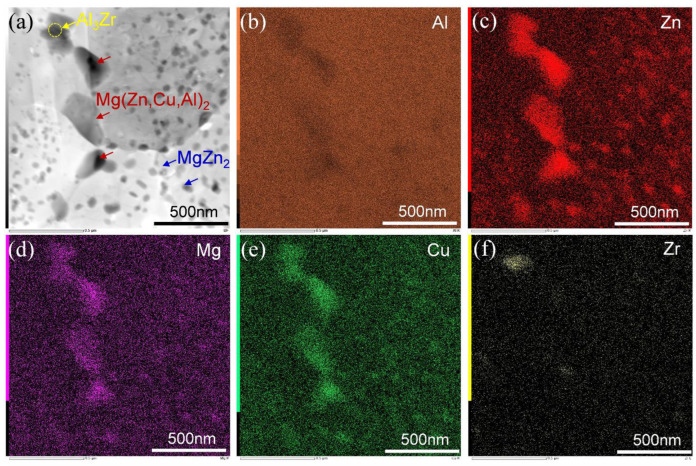
STEM image and nanoscale EDS mapping of Al, Zn, Mg, Cu, and Zr: (**a**) STEM image; (**b**) distribution of Al; (**c**) distribution of Zn; (**d**) distribution of Mg; (**e**) distribution of Cu; (**f**) distribution of Zr.

Figure 4 shows STEM images of the LHZ and HHZ of the builds in the states of NA-7 d, 120 °C × 24 h, and 100 °C × 48 h. The results indicated that several second-phase particles were precipitated along the grain boundaries and in the grain interiors of the builds even in the NA-7 d state and did not change obviously after the artificial aging at 120 °C and 100 °C. The size of the precipitates in the LHZ (Figure 4a–c) was significantly larger than those in the HHZ (Figure 4d–f). To confirm the components of these second-phase particles, STEM-EDS mapping was conducted and the corresponding mapping results are shown in Figure 5. The irregular particles with a size of 200–500 nm distributed along the grain boundaries contained Al, Zn, Mg, and Cu elements. Based on Refs. [24,43,44], these coarse particles were Mg(Zn,Cu,Al)_2_ phases, which were easy to precipitate on the grain boundaries, subgrain boundaries, and some dispersoids during the FSAM process of Al–Zn–Mg–Cu alloys, and primarily deteriorated the mechanical properties of the build. Furthermore, MgZn_2_ phases with a size of 50–100 nm also precipitated along the grain boundaries and inner grains. Apart from the Mg(Zn,Cu,Al)_2_ phase and MgZn_2_ phase, the enrichment of the Zr element was also observed, implying the existence of the Al_3_Zr dispersoid, which can provide the nucleation sites for the precipitates.

**Figure 6:**
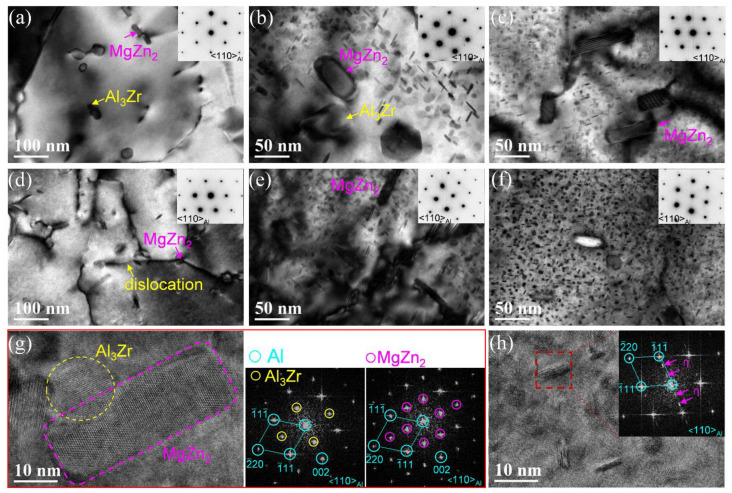
TEM and HRTEM images of the precipitates in LHZ and HHZ of the builds in the different aging states: (**a**) LHZ in the NA-7 d state; (**b**) LHZ in the 120 °C × 24 h state; (**c**) LHZ in the 100 °C × 48 h state; (**d**) HHZ in the NA-7 d state; (**e**) HHZ in the 120 °C × 24 h state; (**f**) HHZ in the 100 °C × 48 h state; (**g**) HRTEM and FFTs of Al_3_Zr and MgZn_2_ phases; and (**h**) HRTEM and FFT of η′ phase.

To further confirm the characteristics of the nanoscale precipitates in the states of NA-7 d, 120 °C × 24 h, and 100 °C × 48 h, the magnified TEM and HRTEM were used to observe the microstructures at LHZ and HHZ of the builds. The electron beam was along <110>_Al_. In addition to the coarse MgZn_2_ phases, few fine strengthening precipitates were precipitated in the NA-7 d state, as shown in Figure 6a,d. However, after artificial aging at 120 °C for 24 h (Figure 6b,e), a large number of nanoscale particles precipitated in the matrix. Additionally, these precipitates were of a smaller size and higher density in the state of 100 °C × 48 h (Figure 6c,f). HRTEM and FFT were used to analyze these nanoscale precipitates, and it was discovered that some of these particles were stable η (MgZn_2_) phases precipitated near the Al_3_Zr particles. As shown in Figure 6g, the clear spots at 1/2 {002}_Al_ and 1/2 {220}_Al_ were from Al_3_Zr particles, and the diffraction spots at 1/3 and 2/3 {220}_Al_ were from MgZn_2_ phases. Furthermore, Figure 6h shows that the metastable η’ phase precipitates in the matrix and their diffraction streaks were parallel to the <111>_Al_ direction in the FFT image. In addition, the η’ phases were the main strengthening precipitates of the build in the state of 100 °C × 48 h.

## 4. Discussion

For the heat-treatable aluminum alloy build, the precipitate characterization is a crucial factor that affects the mechanical properties of the FSAMed build. In this study, the hot-rolled BMs after solution treatment with no obvious precipitates were used to conduct the underwater FSAM. However, after the underwater FSAM, several coarse Mg(Zn,Cu,Al)_2_ and MgZn_2_ phases precipitated even in the natural aging state. These particles tended to form preferentially at grain boundaries, subgrain boundaries, dislocations, and dispersoids because these heterogeneous nucleation sites have high interfacial energies, and the grain boundaries, subgrain boundaries, and dislocations are rapid diffusion channels for solute atoms [45]. During the high-temperature deformation process, Zn, Mg, and Cu atoms in the solid solution tended to segregate at grain boundaries and formed Mg(Zn,Cu,Al)_2_ and MgZn_2_ phases, and then grew rapidly and finally exhibited a large size [46,47]. Additionally, the size of these coarse Mg(Zn,Cu,Al)_2_ and MgZn_2_ phases in the LHZ of the build was higher than those in the HHZ, which was mainly attributed to the finer grains and higher density of subgrains, and dislocations appeared in the LHZ [19]. These finer grains and higher density of subgrains and dislocations facilitated the precipitation and growth of these particles in the LHZ, thereby reducing the solidification degree and the aging strengthening ability of the LHZ [48,49]. Therefore, the microhardness and strength of the LHZ were lower than those of the HHZ after artificial aging. Furthermore, these microhardness fluctuations were not suppressed even after the low-temperature artificial aging, because these coarse Mg(Zn,Cu,Al)_2_ and MgZn_2_ phases already precipitated in the as-fabricated state.

These fine grains, high density of dislocations and substructures, and Al_3_Zr particles not only promote the precipitation of the coarse Mg(Zn,Cu,Al)_2_ and MgZn_2_ phases but also facilitate the precipitation of the nano-scale strengthening precipitates during the artificial aging process [50,51]. If a high-temperature aging treatment is applied to the build, the strengthening precipitates are prone to coarsen during the aging process, resulting in over aging. As illustrated in Figure 6b,e, a more stable η phase precipitated in the build after aging at 120 °C. However, the metastable η’ phases were the main strengthening precipitates of the build in the state of 100 °C × 48 h (Figure 6c,f). Therefore, the microhardness and tensile properties of the build after aging at 100 °C for 48 h were significantly higher than those of the build after aging at 120 °C × 24 h.

As in the abovementioned analysis, the low-temperature aging treatment can effectively avoid the over-aging problem during the post-fabricated aging process and enhance the mechanical properties of the build even without solution treatment. Furthermore, considering the efficiency issue, the aging temperature cannot be too low, because the lower the temperature, the longer the time to achieve a certain strength.

## 5. Conclusions

In this study, the influence of post-fabricated aging treatment on the microstructure and mechanical properties of the Al–7.5 Zn–1.85 Mg–1.3 Cu–0.135 Zr alloy build fabricated by the underwater FSAM was investigated. The following are the main conclusions:

(1) Numerous coarse Mg(Zn,Cu,Al)_2_ and MgZn_2_ phases precipitated along the grain boundaries, subgrain boundaries, dislocations, and Al_3_Zr particles in the build during underwater FSAM. The number of these coarse particles in the LHZ was higher than that in the HHZ, resulting in a lower aging strengthening ability in the LHZ.

(2) Over-aging occurred in the LHZ after artificial aging at 120 °C for 24 h because the high density of grain boundaries, subgrain boundaries, dislocations, and Al_3_Zr particles facilitated the precipitation.

(3) Low-temperature aging treatment can effectively avoid the over-aging problem, significantly enhancing the mechanical properties of the build. After aging at 100 °C for 48 h, the average microhardness value of the build reached 178 MPa; the YSs of the LHZ and HHZ were 453 MPa and 463 MPa, respectively; and the UTSs of the LHZ and HHZ increased to 504 MPa and 523 MPa, respectively.

## Figures and Tables

**Table 1:** Chemical composition of the base metal used in this study (weight %).

Zn	Mg	Cu	Zr	Cr	Ti	Fe	Si	Al
7.50	1.85	1.30	0.135	0.019	0.056	0.083	<0.0002	Bal.

**Table 2:** Tensile properties of the typical Al–Zn–Mg–Cu alloys and the builds fabricated by different additive manufacturing.

Sample	YS/MPa	UTS/MPa	EL/%	Ref.
This study (AA-100 °C × 48 h)	453~463	504~523	13.4~13.6	
7085-T7451	487	509	-	[38]
7085 (AA-150 °C × 8 h)	489	542	13.0	[39]
WAAM 7055 (As-fabricated)	148	231	3.2	[6]
WAAM 7050 (As-fabricated)	-	256	6.3	[40]
WAAM Al-Zn-Mg-Cu (T6)	270~280	415~425	8.5~11.5	[7]
SLM 7075 (As-fabricated)	397	446	6.5	[41]
SLM 7050-T74	449~464	495~505	7.3~7.5	[42]

## Data Availability

The raw/processed data required to reproduce these findings cannot be shared at this time, as the data also comprise a part of an ongoing study.
